# Proceedings of the twenty-second International Society of Sports Nutrition (ISSN) Conference and Expo; Guest Editors: Guillermo Escalante and Chad Kerksick

**DOI:** 10.1080/15502783.2025.2550138

**Published:** 2025-08-30

**Authors:** Guillermo Escalante, Chad M. Kerksick

**Affiliations:** a California State University, Department of Kinesiology, San Bernardino, USA; b Lindenwood University, Exercise and Performance Nutrition Laboratory, Kinesiology Department, College of Science, Technology, and Health, St. Charles, MO, USA

**Keywords:** Abstracts, dietary supplements, nutrition, sports nutrition, health, performance

The 22nd Annual International Society of Sports Nutrition (ISSN) Conference was recently held on June 23–25th in Delray Beach, Florida. This event marks another important milestone in the Society’s continued mission to advance the science and application of sports nutrition as over 350 attendees converged on the Delray Beach area along with a record-setting number of research abstracts. As has become expected of this event, the 2025 meeting brought together a global community of researchers, clinicians, students, industry professionals, and thought leaders dedicated to enhancing human performance and health through evidence-based nutritional strategies.

This year’s meeting featured over 70 original research abstracts spanning a wide range of innovative topics including ergogenic aids, body composition, hydration, energy systems, recovery, performance enhancement, probiotics, protein supplementation, and nutritional strategies. We also saw an increased emphasis on personalized nutrition, female-specific research, and translational approaches bridging laboratory findings with real-world athletic and clinical applications.

We were privileged to host Dr. Michael Roberts, who holds an Endowed Professor position at Auburn University for a keynote lecture where he presented his work on skeletal muscle hypertrophy. Dr. Matt Frakes, Performance Nutrition Director for the New York Giants, also joined us and shared with us many of the things that make him one of the best sports dietitians in the sport of football. In addition to these impactful talks, the conference provided a platform for early-career researchers and students to showcase their work, connect with established professionals, and spark collaborations that will shape the future of sports nutrition.

The ISSN continues to promote a forward-thinking environment that values diversity in scientific inquiry, methodology, and participant populations. We believe this approach enhances the quality and applicability of our field and encourage its continued growth. Delray Beach provided an ideal backdrop for scientific exchange and professional networking. The coastal setting not only inspired reflection and connection but reminded us of the importance of balancing high-impact work with high-effort play.

As leaders of the ISSN scientific program for this year’s meeting, we are pleased to present this supplement of research abstracts as a representation of the exciting and impactful science presented at ISSN 2025. These abstracts reflect the dynamic growth of the field, the innovative spirit of its contributors, and the unwavering commitment of the ISSN community to elevate the science of sports nutrition.

We thank all speakers, exhibitors, sponsors, attendees, and volunteers who made this year’s conference a resounding success. We hope you find inspiration in the work shared and that the discussions and connections formed in Delray Beach will energize your own professional journey.

We look forward to seeing you at next year’s meeting June 17 - 19th in Ft. Lauderdale, FL, and continuing the momentum built here in 2025.

